# 

**Published:** 1907-11-23

**Authors:** 


CONTENTS.

| | PAGE |
|---|---|
| **The Significance of Persistory Pyrexia** | 197 |
| **Hospitals and Suicidal Patients** | 198 |
| **Annotations—** | |
| The Early Days of Listerism—Society for the Study of Disease in Children—Cancer Infection and Cancer Recurrence | 199 |
| **Medical Opinion and Movement** | 200 |
| **Hospital Clinics—** | |
| The Importance of Localising the Heart's Apex Beat. By Arthur Foxwell, M.A., M.D.Cantab., F.R.C.P. | 201 |
| Brachial Neuritis and Similar Conditions Involving the Arms. By T. D. Savill, M.D.Lond. | 203 |
| **Special Article—** | |
| Temperature Variations in Pulmonary Tuberculosis | 207 |
| **Points in Diagnosis—** | |
| The Bismuth Skiagram in Investigating Gastro Intestinal Disorders | 209 |
| **Points in Pathology—** | |
| Acetonæmia and Acetonuria | 210 |
| **Points in Surgery—** | |
| The Treatment of Fractures from a Common-sense Point of View | 211 |
| Epithelioma of the Penis | 212 |
| **The General Practitioner's Column—** | |
| Sanatorium Experiences | 213 |
| **Laryngology and Rhinology—** | |
| Local Applications to the Upper Air Passages | 215 |
| **The Book World of Medicine and Science** | 216 |
| **Dermatology—** | |
| Some of the Newer Dermatological Remedies and their Value | 217 |
| **Practical Notes on Diagnosis and Treatment** | 218 |
| **New Appliances and Things Medical** | 218 |
| **Hospital Administration—** | |
| Hospitals for the Paralysed | 219 |
| Medical Students as Nurses—II. | 220 |
| The Graduates' Medical Schools | 221 |
| The Medical Graduates' College and Polyclinic | 223 |
| **Nursing Administration—** | |
| The Registration of Nursing Homes | 222 |
| **Editor's Letter-Box—** | |
| "Professional Freedom" | 223 |
| The Royal Free Hospital | 223 |
| The late Mr. Reginald Lund | 223 |
| Diseases of Twins | 224 |
| The "Referee" Children's Dinner Fund | 224 |

